# Food environment in and around schools and colleges of Delhi and National Capital Region (NCR) in India

**DOI:** 10.1186/s12889-021-11778-6

**Published:** 2021-09-28

**Authors:** Shalini Bassi, Deepika Bahl, Monika Arora, Fikru Tesfaye Tullu, Sakshi Dudeja, Rachita Gupta

**Affiliations:** 1 Public Health Foundation of India, Plot No. 47, Sector 44, Gurgaon, Haryana 122002 India; 2 World Health Organization, Country Office for India, RK Khanna Tennis Stadium, Safdarjung Enclave, New Delhi, India

**Keywords:** Adolescents, Children, Obesity, Schools, Colleges, Canteen policy, Food environment

## Abstract

**Background:**

Food policies and environment (availability, accessibility, affordability, marketing) in and around educational institutes can influence food choices and behaviours of children and adolescents.

**Methods:**

Cross-sectional, mixed-methods study was implemented in schools (*n* = 9; Private = 6, Public = 3) and colleges (*n* = 4) from Delhi and National Capital Region (India). The data was collected from students of schools (*n* = 253) and colleges (*n* = 57), parents of school students (*n* = 190), teachers (*n* = 12, schools = 9, colleges = 3) and canteen operators of Private schools and colleges (*n* = 10; schools = 6, and colleges = 4). The primary and secondary data was collected to: 1) identify the strengths and weaknesses of the existing guidelines and directives (desk review); 2) examine food environment, existing policies and its implementation (structured observations, in-depth interviews, surveys, focus group discussions), and; 3) assess food choices, behaviours of students (focus group discussions). The thematic analysis was used for qualitative data and descriptive analysis for quantitative data.

**Results:**

The available food and beverage options, in and around the participating educational institutes were either high in fat, salt and sugar (HFSS), despite government and educational institute guidelines on restricting the availability and accessibility of HFSS foods. The healthy food and beverage options were expensive compared to HFSS foods both inside and outside educational institutes. In total, 37 vendors (Private = 27; Public:10) were observed outside schools at dispersal and twelve at lunchtime. Around colleges, vendors (*n* = 14) were seen throughout the day. Students from all Private schools (*n* = 6) and colleges (*n* = 2) were exposed to food and beverage advertisements either HFSS (Private schools = 1–3 and colleges = 0–2 advertisements), whereas no advertisements were observed around Public schools.

**Conclusion:**

It is imperative to implement food policies to improve the food environment in and around educational institutes to ensure the availability of healthy foods to establish and sustain healthy eating behaviours among students. Thus, the study findings emphasise stringent implementation, regular monitoring and surveillance of recently introduced Food Safety and Standards (Safe food and balanced diets for children in school) Regulation 2020, ensuring its compliance through effective enforcement strategies.

**Supplementary Information:**

The online version contains supplementary material available at 10.1186/s12889-021-11778-6.

## Background

Overweight and obesity among children elevate the risk of non-communicable diseases (NCDs) in adulthood [1]. In all, 5% of NCDs are associated with obesity and overweight [2]. Children who are overweight and obese are more likely to develop high blood pressure, high cholesterol, insulin resistance, respiratory and joint problems [3]. Obesity has an impact on children’s physical, psychological health as well as on, social behaviour [3].

Globally, overweight and obesity among children has increased substantially in recent decades [4], with a similar trend in India. The Comprehensive National Nutrition Survey report (2016–18) revealed that 4.9% of boys and 4.7% of girls aged 10–19 years are either overweight or obese (Body Mass Index for age; z-score > + 1 SD). Obesity was also observed to be higher in urban areas (9.7%) than in rural (3.2%). Judging by the wealth quintile, the prevalence of obesity was lowest among the poor (0.8%) and highest among rich (11.6%) [5].

The complex web of factors that include the availability of energy-dense, nutrient-poor foods, and the presence of child-oriented marketing contribute majorly to unhealthy food consumption patterns in children [6–9]. As it is seen that children spend much of their daytime in and around the school hence, a supportive food environment is critical in shaping a child’s overall development and strongly impacting their food intake and weight status [10–13]. The transition to college life is critical when adolescents make all food decisions independently [14]. In India, various interventions have been implemented among children and adolescents to improve their knowledge, attitude, and behaviours to combat overweight and obesity [15, 16]. But less focus has been given to assess the food environment of educational institutes in India.

Healthy food policies and environment in educational institutes influence food behaviours and practices of students thereby improving their nutritional status, academic achievement, and health in adulthood [15]. In India, various guidelines and directives have been issued to restrict the availability and accessibility of foods high in fat, salt, and sugar (HFSS) to students in and around their educational institutes. These draft guidelines and directives aim to improve the overall food environment in and around schools and colleges. These guidelines and directives have been issued by the Ministry of Women and Child Development [16], Food Safety and Standard Authority of India (FSSAI) [17, 18], Central Board of Secondary Education [19], and University Grant Commission [20]. Recently, Food Safety and Standards (Safe food and balanced diets for children in school) Regulation, 2020 was also introduced by FSSAI to ensure the availability of safe and balanced diet to school children [21].

Globally, enough evidence is available on the obesogenic food environment in and around schools and colleges and its impact on overweight and obesity. However, this evidence is lacking in India. We, thus, aim to identify the strengths and weaknesses of the existing guidelines and directives issued by the concerned government authorities on food environment (availability, affordability, accessibility and food marketing) in and around educational institutes (schools and colleges). We also assessed food choices, behaviour and practices of school and college students.

## Methods

### Study design and setting

**Fig. 1:**
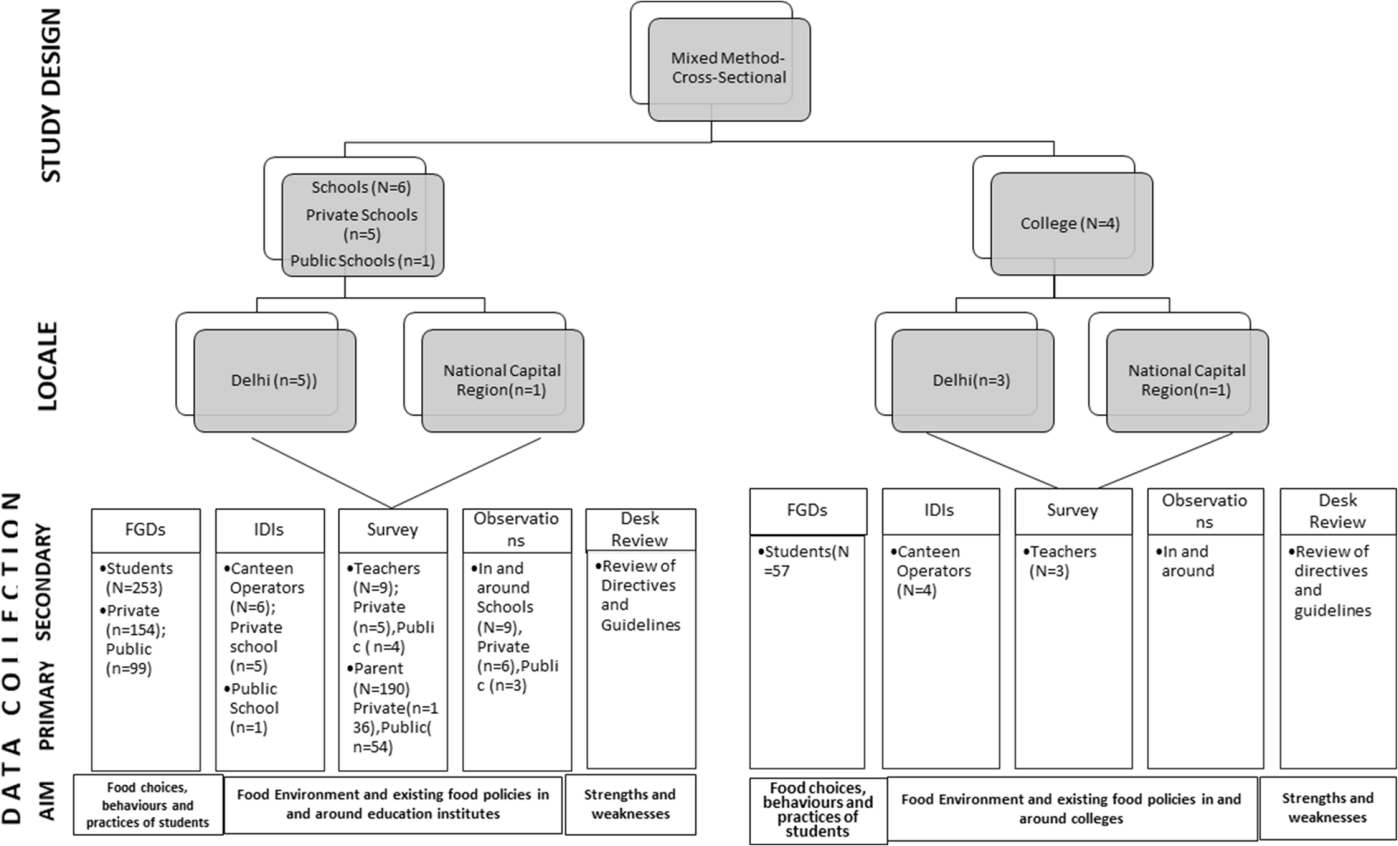
Study Design

A cross-sectional, mixed-methods study was conducted from June till November, 2019 in nine schools and four colleges situated in Delhi and National Capital Region (NCR). Six Private (Delhi = 5, NCR:1), six Public schools (*n* = 3; Delhi = 3) and 4 colleges (Delhi = 3, NCR = 1) were included in the study (Fig. 1). A mixed-method approach was used to examine the food environment, existing food policies, and to assess food choices, behaviours, and practices of school and college students. Delhi is included in the present study as the prevalence of overweight (12.3%) and obesity (3.3%) among adolescents (10–19 years) in Delhi is among the top three states in the country. Delhi is a metropolitan city in North India and is surrounded by neighbouring cities (Ghaziabad, Faridabad, Gurgaon and Noida) in an area called the NCR [22].

### Sampling and participants

The purposive sampling was used to recruit schools, colleges, and study participants (students, teachers, canteen operators and parents). The list of schools available on the Directorate of Education’s website was referred to select Private and Public schools [23]. From NCR, Private schools and colleges were recruited from existing collaborations of authors. In India, Private schools are administered by a private body and receive no government funding, whereas Public schools are managed and receive funding from the government and affiliated bodies [24]. Public schools mainly cater to students from low socio-economic status while, Private schools cater to students from middle to high socio-economic status. The school type (Private and Public) was used as a proxy measure for socio-economic status in accordance with earlier similar studies from India [25, 26]. Inclusion criteria for the study were, co-educational schools (Private and Public) with primary (I-V grades) up till secondary (X grade) level education and a Private school with a canteen. Similarly, colleges affiliated with University Grant Commission and having a canteen were eligible for inclusion.

A written informed active consent from the participating schools (Private and Public), colleges, college students, teachers, canteen operators and parents were taken. In the case of minors (< 18 years), a written informed assent from a student and a consent from their parent or guardian was also obtained.

### Data collection

The data was collected using both primary and secondary methods to: 1) identify the strengths and weaknesses of the existing guidelines and directives, 2) examine food environment, existing policies and their implementation, 3) assess the food choices, practices and behaviours of students. The primary data was collected using in-depth interviews (IDIs) with canteen operators, surveys with teachers and parents of school students, observations in and around schools and colleges (during lunchtime and at school/college dispersal) and focus group discussions (FGDs) with school and college students (all study tools provided as Supplementary file 1). All the study tools were pre-tested before the commencement of data collection to ensure contextual relevance, feasibility, and face validity. Under secondary data, a desk review was conducted by collating existing guidelines and directives issued by the concerned government authorities. A comparative analysis was undertaken of these guidelines and directives to identify their strengths and weaknesses.

IDIs were conducted with canteen operators (*n* = 10; schools = 6, colleges = 4) to understand the existing school and college canteen policies and their implementation. A self-administered survey was conducted with school and college teachers (*n* = 12; schools = 9, colleges = 3) and parents of school children (*n* = 190/253), to capture their views on the current food policies and environment. For teachers, a copy of the survey was personally given by the study team, whereas for parents, the survey was sent home with students and later, the study team collected from students.

The structured observations (*n* = 13; schools = 9, college = 4) were conducted in and around participating schools and colleges within a distance upto 200 m. A distance of 200 m was assessed through GPS Field Area Measure [27]. Two researchers conducted these observations independently using the structured checklist to understand the food environment and implementation of existing food policies in and around educational institutes. The data were then reviewed, all discrepancies were resolved in consultation with other authors.

The checklist for observation was developed based on the recommendations by Women and Child Development [16], FSSAI [17, 18], Central Board of Secondary Education [19], University Grant Commission [20], and INFORMAS “Outdoor Advertising: School Zones” [28]. The information collected from teachers, parents, and students was also validated through these observations.

Based on observations and IDIs with the canteen operators, a list of available foods and beverages (packaged and unpackaged/ non-standardised proprietary foods) was prepared. The available packaged food and beverage options were categorised into healthy or HFSS, using WHO’s Nutrient Profile Model for South-East Asia Region (SEAR). This model is based on thresholds for total fat, total sodium, and total sugar. According to this model, the sodium threshold is 1 mg sodium:1 kcal energy or lower, sugar threshold is equal to or higher than 10% of the total energy (kcal) for the product of fat is equal to or higher than 30% of the total energy (kcal) [29]. This model was only used for the processed packaged foods and beverages because of the labelling information available on these foods and beverages.

Similarly, there are existing guidelines [16, 17] and directives [20] that also specify the categorisation of non-standardised proprietary/unpackaged and packaged foods and beverages based on the colour (green, yellow and red) in accordance to their nutritional value [16, 18]. These guidelines were then utilised to classify foods and beverages available in and around the participating educational institutes.

The FGDs, using semi-structured guide were conducted with students (*n* = 32 FGDs and 310 students; schools = 25 FGDs, 253 students; colleges = 7 FGDs, 57 students) to understand their food choices, behaviour, practices and influence of food and beverage advertisements on them.

All the FGDs (with 8–10 students), IDIs and teacher’s surveys were conducted inside the school and college premises after taking prior approval from the concerned authorities. An overview of data collection methods, participants and themes has been presented in Table 1.

**Table 1:** An overview of qualitative and quantitative data collection methods, participants and themes

Theme	Data collection method used and target group				
	IDI with canteen operators	FGDs with students	Surveys with teachers	Surveys with parents of school students	Observations (in and around educational institutes)
Nutritional policy or guidelines for the available foods available in canteen	✓		✓	✓	
Available food and beverage (packaged/unpackaged) options to students in canteen	✓	✓	✓	✓	✓
Restriction on the sale of foods high in fat, salt and sugar in and around schools and colleges	✓	✓	✓	✓	✓
Colour coding for the foods served in canteen	✓		✓	✓	✓
Available food and beverage (packaged/unpackaged) options to students outside educational institutes up to 200 m through vendors		✓			✓
Accessibility of canteen services to students (canteen timings for students)	✓	✓	✓	✓	✓
Vendor access to students		✓	✓	✓	✓
Affordability of available foods and beverages in canteen (pricing guidelines)	✓	✓	✓	✓	
Food advertisements and marketing in and around educational institutes		✓	✓		✓
Influence of food and beverage advertisements (TV, canteen, social media)		✓			
Event sponsorship by food and beverage companies		✓	✓		
Food consumption patterns		✓			
Dietary behaviors of students		✓			
Food choices and preferences		✓			
Eating out (canteen, vendors, restaurant)		✓			
Factors contributing for the consumption of foods high in fat, salt and sugar		✓			

### Data analysis

Quantitative data from the surveys that were administered with parents and teachers and the observations made in an around school premises was cleaned, entered (ACCESS software), and analysed using SPSS version 22 (IBM Corp., Armonk, NY, USA). Descriptive statistical measures were used to analyse quantitative data. For qualitative data, all the audio recordings from FGDs and IDIs were transcribed in Hindi (the local language) [30] and translated into English for inductive thematic analysis.

For policy review, two authors independently (SB and DB) reviewed the variables enlisted in the guidelines, directives, and regulations to identify their strengths and weaknesses. Any disagreement between the two authors (SB and DB) was further resolved by a discussion with the third author (MA).

## Results

This section describes the findings that are derived by reviewing the existing guidelines and directives issued by the concerned authorities, also discuss the food environment (in and around participating schools and colleges) and perspective of multiple stakeholders on existing food policies in schools and colleges. Students’ perception regarding their food choices, behaviour, practices and the effect of food advertisements on them is also highlighted in this section.

The overall response rate of the parent survey was 75.1% (Private: 61% and Public: 38%). The reasons for non-participation included hesitancy in sharing the information, poor literacy levels, and lack of time to fill the survey (as reported by the students).

The determinants of food environment covered the following aspects: availability (foods and beverages available to students in and around their schools and colleges), accessibility (canteen timings, vendor access to students), affordability (pricing of available foods and beverages), marketing (foods and beverages advertisements).

### Review of existing guidelines and directives issued by the concerned authorities

A review of existing guidelines, directives [16–20], and regulations [21] (Table 2) highlighted that all of these guidelines and policies aimed at improving the overall school and college canteen environment in terms of availability, accessibility of healthy food and beverage options and restricting the marketing of HFSS foods and beverages to students. The majority of these guidelines and directives emphasised on enhancing the health of school children leaving college students behind. None of these guidelines and directives mentioned pricing, which can encourage the consumption of healthy foods, beverages and curb the consumption of HFSS foods and beverages. The majority of these guidelines do not highlight the restriction of hours granted to access canteen services by students. Colour coding of the available foods and beverages (packed and unpacked) in the school canteen has been recommended by two guidelines, without specifying the nodal person responsible for its monitoring. There is a lack of clarity on the implementation of these guidelines, making it difficult for various stakeholders to enforce them and ensure accountability. Information on portion size has been specified in terms of “right portion size,” but guidelines lack the operational definition of the “right portion size”. According to all the guidelines and directives, there should be no sale of HFSS foods and beverages in and around the educational institution. Although none of them specifies the punitive actions that can be taken in case of any sale of HFSS foods and beverages to the students. The recently notified Food Safety and Standards (Safe food and balanced diets for children in school) Regulation, 2020 [21] by FSSAI ensures the availability of safe and wholesome food in school premises. It encompasses various aspects to protect and conserve the school food environment, including the promotion of safe food and balanced diet in and around the school campus and also restrictions of advertisements, marketing, and selling of HFSS foods and beverages on the school campus or to school children in an area within fifty meters from the school gate (in any direction) and regular monitoring and surveillance mechanism through designated authorities, corporations, and committees.

**Table 2:** Policy review of existing guidelines, directives and regulations

		WCD, 2015 [16]	FSSAI, 2015 [17]	CBSE, 2016 [19]	UGC, 2018 [20]	FSSAI, 2019 [18]	FSSAI, 2020 [21]
S. No	**Aim of the guideline, directive and regulation**	Addressing the consumption of HFSS foods and promotion of healthy snacks in schools of India	Guidelines for making wholesome, nutritious, safe and hygienic foods to school children in India	Promotion of healthy snacks in schools affiliated to CBSE	To ban junk food in colleges and develop a new standard for healthy foods and make student life better. To reduce the prevalence of obesity and comorbidities such as lifestyle-related diseases	Draft Notification on Food Safety Standards (Safe Food and Healthy Diets for School Children) Regulation, 2019	Food Safety and Standards (safe food and balanced diet for children in school) Regulation, 2020
1)	**Target Population**	School students	School students	School students	College students	School students	School students
2)	**Canteen policy**						
a)	Colour coding of foods to green, yellow and red	√	√	×	×	×	×
b)	80% of available food in school should be of green category	×	√	×	×	×	√
c)	School Canteen Management Committee	√	√	√	×	√	√
3	Restriction on availability of HFSS foods in an institute	√	√	√	√	√	√
4	Restriction on availability of most common HFSS foods in the nearby area	200 m	50 m	200 m	×	50 m	50 m
5	Shops and restaurants selling proprietary foods within the vicinity of 200 m of a school should not be permitted to sell these foods to school children in uniform.	√	×	×	×	×	×
6	Specification on portion size	√		×	×	√	Only for desserts, packaged foods, bakery products and beverages
7.	Marketing and advertisement of HFSS foods to children *No offer or free of sale of HFSS foods to children in an institute and around 50 m*	×	×	×	×	√	√
8.	No logos, brand names, spokes character, product names, other product marketing on/in vending machines, etc.	×	×	×	×	√	√
9	Existence of display board restricting sale of HFSS foods inside the school premises	×	×	×	×		√

(√): if an instruction is given for a specific measure; (×): if an instruction is lacking for a specific measure

*WCD* Ministry of Women and Child Development, *FSSAI* Food Safety and Standard Authority of India, *CBSE* Central Board of Secondary Education

### Food environment and policies

#### Availability of food and beverage options in and around participating schools and colleges

Observations and interactions with the study participants (students, teachers, parents and canteen operator) showed that a variety of food and beverage options were available in canteens of the participating Private schools and colleges. According to discussions with students, the most frequently available packed and non-standardised proprietary (unpackaged foods and beverages) options were samosa (fried pastry with potato filling), burgers, potato chips, pizza, vegetable puff, potato cutlets, bread pakoda (bread fritters), french fries, ice-creams (milk-based and ice candies), sugar-sweetened carbonated beverages and sugar-sweetened non-carbonated beverages (e.g. packed juices with sugar). The available healthy options in the canteen were cereal-pulse combinations [fermented e.g. idli/uttapam with sambhar, rice and pulse combinations and non-fermented e.g. rajma rice (beans curry with rice), chole rice (chickpea curry with rice)], vegetable-cereal combination e.g., vegetable parantha (vegetable stuffed flatbread) and variety of beverages (lemonade without sugar, fresh seasonal fruit juices).

The majority of parents (92.6%) and all the canteen operators of schools reported the availability of HFSS/(red category foods and beverages more than green category/healthy foods and beverages) (Table 3). In contrast, teachers reported having school policies in place that restrict the sale of HFSS/red category foods. All Private schools (*n* = 6/6) reported restricting the sale of sugar-sweetened beverages (carbonated), two Private schools banned the sale of fried foods, and only one Private school banned the sale of food items high in salt. However, observations made by the study team reported the availability of these food and beverage items in the school canteen. Despite restrictions, the sale of sugar-sweetened beverages (carbonated) was observed in one Private school (Table 3). Among colleges, only one college in NCR had the policy to restrict the sale of sugar-sweetened beverages (carbonated). Apart from this, no other college had any policy restricting the sale of HFSS foods. There was no difference in the observations made by the study team and that reported by the canteen operators about the availability of healthy and HFSS foods in both schools and colleges, but for beverages, a difference was noted. Canteen operators reported higher availability of healthy drinks, but observations showed less availability of healthy beverages in schools (Table 3).

**Table 3:** Availability of foods and beverages (packaged and unpackaged) in school^a^ and college canteen

Colour coding categories for foods and beverages^b^		Private Schools (***N*** = 6)			Colleges (***N*** = 4)	
		Parents (***N*** = 118)	Canteen Operator-Schools (***N*** = 6)	Observations (***N*** = 6)	Canteen Operator-Colleges (***N*** = 4)	Observations (***N*** = 4)
		N (%)	N (%)	N (%)	N (%)	N (%)
**Food items**						
Green	Cereal and pulse combos (beans curry with rice/chickpeas curry with rice), vegetable sandwiches, fermented items (rice and pulse steamed cakes), fruits and vegetables	96 (70.6)	5 (83.3)	5 (83.3)	4 (100)	4 (100)
Yellow	Ice-creams (milk-based)	Not reported	2 (33.3)	2 (33.3)	4 (100)	4 (100)
Red	Samosa (fried pastry), vegetable puffs, candies, chocolates, cookies, instant noodles, burgers, french fries, bread fritters, ice-cream (ice candy)	126 (92.6)	6 (100)	6 (100)	4 (100)	4 (100)
**Beverages**						
Green	Coconut water, fresh fruit juice, lemonade without sugar	67 (49.3)	2 (33.3)	1 (16.6)	2 (50)	2 (50)
Yellow	Flavoured milk	22 (16.2)	3 (50)	4 (66.6)	3 (75)	3 (75)
Red	Sugar Sweetened Beverages (carbonated)	41 (30.1)	1 (16.6)	1 (16.6)	4 (100)	4(100)
Red	Sugar Sweetened Beverages (Non-carbonated) e.g. Fruit juices with sugar	69 (50.7)	6 (100)	6 (100)	4 (100)	4 (100)

^a^Only data from Private schools, as none of the Public schools had a canteen

^b^Foods and beverage classification based on “Guidelines for making wholesome, nutritious, safe and hygienic foods to school children in India”, FSSAI 2015 [17]

The majority of parents (77.6%) expressed that foods in the canteen should be colour coded (like green, yellow, and red) to encourage children to make healthy food choices. Only one out of ten canteen operators in schools and colleges were aware of the colour coding concept. None of the canteens of Private school (*n* = 6) and college (*n* = 4) displayed foods and beverages, based on three colour coding categories. They also did not have any instructions from the authorities or management to follow the same.

The foods and beverages were also available outside schools and colleges through vendors. Vendors were selling food items like ice-creams (milk-based and ice-candies), candy floss, fried snacks, churan (sugar balls*),* instant noodles, cookies, salted savoury snacks, and tea (Table 4). It was also observed that more Public schools (2 out of 3) in comparison to Private schools (2 out of 6) had vendors selling green category food (for e.g.: boiled chickpea salad with flatbread) in accordance with the colour coding guidelines for non-standardised foods/unpackaged foods. Whereas the scenario in college was much better, most vendors outside the colleges (3 out of 4) were selling healthy food options such as composite meals (made of a combination of food groups like cereal and pulse). (Table 4).

**Table 4:** Availability of foods and beverages (packaged and unpackaged) outside schools and colleges through vendors

Colour coding categories for foods and beverages^a^		Observations		
		Private-schools n (%)	Public schools n (%)	Colleges n (%)
		*N* = 6	*N* = 3	*N* = 4
**Food items**				
Green	Matra kulcha (Flat Breads with boiled chickpea salad)	2 (33.3)	2 (66.6)	3 (75)
Yellow	Ice-creams (milk-based)	6 (100)	3 (100)	4 (100)
Red	Fried snacks with potato curry, sugar balls, instant noodles, candy floss, salted savoury snacks, cookies, chips, candies, −ice-creams (ice candies)	6 (100)	3 (100)	3 (75)
**Beverage items**				
Yellow	Tea	5 (83.3)	3 (100)	4 (100)
Red	Carbonated sugar sweetened beverages	1 (16.6)	2 (50)	4 (100)

^a^Foods and beverage classification on the basis of guidelines for making wholesome, nutritious, safe and hygienic foods to school children in India, FSSAI 2015 [17]

The available ‘packed foods and beverages’ being sold in the canteen and around the participating schools and colleges were analysed using WHO’s Nutrient Profile Model for SEAR [29], and it showed that all available packed foods and beverages were either HFSS (Table 5).

**Table 5:** Categorization of available packaged foods in and around schools and colleges based on WHO’s Nutrient Profile Model for SEAR

Packed foods & beverages	Total Calories (per 100 g/100 ml) (Kcals)	Total sugar/100 g (g)	Total fat/ 100 g (g)	Total Salt (mg)	Categorization of HFSS foods based on thresholds
**Food items**					
Chips (Salted)	550	1	34.3	642	Salt and fat are higher than thresholds
Ice cream –Choco vanilla	290	15.2	17.1	NA	Sugar and fat are higher than thresholds
Biscuits	489	16.1	21.2	NA	Sugar and fat are higher than thresholds
Fiber-rich biscuits	443	22	11	11.3	Sugar higher than thresholds
Honey oat biscuits	485	35	20	275	Fat and sugar are higher than thresholds
Packed noodles	437	3.4	15.7	1232.2	Salt and fat are higher than thresholds
**Beverages**					
Lemonade	45	10.5	0	79	Sugar and salt higher than thresholds
Sugar-sweetened beverage (Carbonated)	44	11	0	0	Sugar higher than thresholds
Juice (Litchi)	60	15	0	0	Sugar higher than the thresholds
Mix fruit juice (Non-carbonated)	56	13.7	0	4	Sugar higher than thresholds
Buttermilk	26	0	1.1	30	Salt and fat higher than thresholds

#### Accessibility of canteen services and vendors around schools and colleges

Teachers from all six Private schools and parents (85.8%) of Private schools reported that students were only allowed to avail canteen service during lunchtime, indicating restriction on accessing the canteen services. But the interviews conducted with canteen operators and the observations made, showed that students were purchasing foods and beverages from the canteen other than lunchtime as well. For colleges, data from students, teachers, canteen operators, and observations highlighted no time restriction for accessing the canteen services by the students.

Children also had easy access to foods and beverages through vendors both during lunchtime and at dispersal. During lunchtime, twelve vendors were observed around six schools (Private: 2 and Public: 4) within a 50 m distance from the school boundary (0–2 vendors around Private and 1–3 around Public school). At the time of dispersal, additional vendors (*n* = 15) were seen outside the schools, making it to a total of thirty-seven vendors at the time of school dispersal. Of these thirty-seven vendors, seventeen vendors were seen within 50 m. The density of these vendors was higher outside the Private schools (*n* = 27) than that of Public schools (*n* = 10) at dispersal. Though vendors increased at the time of dispersal but the type of foods and beverages sold by them were similar during the lunchtime and at dispersal. Around colleges, vendors (*n* = 14) were seen throughout the day within 200 m of the college boundary.

Observations showed that students from Private schools, Public schools and colleges were consuming foods and beverages from these vendors. However, teachers of all schools (Public and Private) specified that there were restrictions and students were not allowed to access food outlets or vendors around the school. One of the teachers from a college also reported that they do not allow vendors to sell foods and beverages outside their college premises.

#### Affordability of available foods and beverages in school and college canteens

Data from IDIs and observations showed that food prices ranged from INR 5 (0.06 USD) to INR 40 (0.53 USD) in school canteens, while INR 10 (0.13 USD) to INR 60 (0.79 USD) in the college canteens. Table 6 highlights that the available healthy food and beverage options (green and yellow category) were expensive in both schools and colleges than HFSS foods and beverages /red category. But according to teachers, pricing guidelines for selling healthy foods and beverages at subsidised prices existed in schools (*n* = 6) and colleges (*n* = 2). Data showed that only one school out of 6 schools capped the pricing for all foods and beverages, irrespective of being healthy or HFSS. They were available at less than INR 15 (0.19 USD). The majority of parents (82.6%) also expressed their inclination towards subsidising healthy foods to encourage healthy eating among children. Prices of foods and beverages outside college premises varied from INR 10 (0.13 USD) to INR 60 (0.79 USD) while it varied from INR 5 (0.06 USD) to 60 (0.79 USD) outside schools (Table 6). The price of healthy food and beverages was higher than HFSS foods and beverages, both outside the schools and colleges.

**Table 6:** Prices of available foods and beverages (packed and unpacked) inside and outside schools and colleges

Colour coding categorization for foods and beverages^a^		Prices in INR (USD)	
		Schools ***N*** = 9	Colleges ***N*** = 4
**Food items (unpacked, in canteen)**			
Green	Cereal and pulse combos (beans curry with rice/chickpeas curry with rice), vegetable sandwiches, fermented items (rice and pulse steamed cakes), fruits and vegetables, flat bread with boiled chickpea salad	15–35 (0.20–0.46)	20–60 (0.26–0.80)
Yellow	Milk ice-creams	20–25 (0.26–0.33)	20–55 (0.26–0.73)
Red	Samosa (fried pastry), Vegetable puffs, candies, chocolates, cookies, instant noodles, burgers, french fries, bread fritters, chips, cookies, sugar balls, candy floss, fried snacks	5–15 (0.06–0.20	10–50 (0.13–0.66)
**Beverages (unpacked, in canteen)**			
Green	Coconut water, fresh fruit juice, lemonade without sugar	10–40 (0.13–0.53)	20–45 (0.26–0.60)
Yellow	Flavoured milk	15–20 (0.20–0.26)	25–35 (0.33–0.46)
Red	Sugar-Sweetened Beverages (carbonated)	15–25 (0.20–0.33)	20–35 (0.26–0.46)
Red	Sugar-Sweetened Beverages (non-carbonated, Fruit juices with sugar)	10–35 (0.13–0.46)	15–25 (0.20–0.33
**Food items (Packed and unpacked, outside schools and colleges through vendors)**			
Green	Matra kulcha (Flat Breads with boiled chickpea salad)	25–60 (0.33–0.79)	35–60 (0.46–0.79)
Yellow	Ice-creams (Milk based)	20–55 (0.26–0.73)	20–55 (0.26–0.73)
Red	Fried snacks with potato curry, sugar balls, instant noodles, candy floss, salted savoury snacks, cookies, chips, ice-creams (ice candies)	5–25 (0.06–0.33)	10–25 (0.13–0.33)
**Beverages (Packed and unpacked, outside schools and colleges through vendors)**			
Yellow	Tea	10 (0.13)	10 (0.13)
Red	Sugar Sweetened Beverages (Carbonated)	20–35 (0.26–0.46)	15–35 (0.20–0.46)

1 USD = 74.81 INR (Indian Rupees)

^a^Foods and beverage classification on basis of guidelines for making wholesome, nutritious, safe and hygienic foods to school children in India,FSSAI 2015 [17]

#### Exposure to food and beverage marketing

Students during discussions reported that food and beverage advertisements on television, in the school canteen, outside school, and on social media platforms does influence their food choices and trigger them to buy the advertised foods. A total of ten food and beverage advertisements were observed across six schools and two colleges (within an area of 200 m from the main entrance). Of these, 1–3 advertisements were observed around Private schools, 0–2 around colleges and no advertisements around Public schools. These advertisements were observed for ice-creams, carbonated sugar-sweetened beverages, ketchup, and frozen foods. The analysis of these packed foods and beverages advertisements using the nutrient profile model classified them to be either HFSS. They were all from the red category when classified according to the colour coding concept. The advertisements were displayed at about 100 to 180 m from the main entrance of the school and college. Out of the ten advertisements, seven were medium sized (> 1.3 m × 1.9 m but < 2.0 m × 2.5 m), two were small in size (>A4 but < 1.3 m × 1.9 m), and one was large (> 2 m × 2.5 m). All the advertisements were displayed as posters either on a shop, or on hawker’s carts or at the bus stops. Additionally, advertisements displaying sugar rich food items (carbonated beverage) were also seen inside the canteen of two colleges, in the form of a poster, which was large in size.

Sponsorship of school and college events by the foods and beverage companies including breakfast cereal, carbonated sugar-sweetened, butter, cookies, chocolate were also reported. The above-mentioned foods were either HFSS, when analysed using the nutrient profile model [29].

### Food consumption patterns, and dietary behaviours of students

Discussions with students and findings from survey with parents highlighted good dietary behaviour of the school students (Private and Public) as they reported consumption of milk and its products, fruits, and vegetables, on a daily basis. Around 90% of school students usually brought lunch from their home on school days, and only 1.1% ate from the school canteen, as reported by their parents. With the availability of HFSS foods and beverages in the canteen, students were more inclined towards them. Students reported purchasing foods and beverages like samosa (fried pastry), vegetable puffs, and sugar-sweetened non-carbonated beverages from the canteen. As reported by parents, more students from Private schools (45.5%) spent their pocket money on eating out than students of Public schools (23.5%). During these discussions, school students (Private and Public) reported eating out with their parents or friends at least once a week and few even reported eating out only once a month on special occasions.

During discussions, it was evident that the consumption of foods and beverage either HFSS by students varied and ranged from once a week to once a month. The frequency of consumption for sugar sweetened beverages was once a week, despite their knowledge about the health consequences of consuming these beverages.

Similarly, college students also preferred eating burgers, sandwiches, and sugar-sweetened carbonated beverages from the canteen. In comparison to school children, along with daily consumption of milk, fruits, and vegetables, college students reported an intake of sugar-sweetened carbonated beverages three to four times a week. Students reported that they consumed sugar-sweetened carbonated beverages due to examination stress, weather, addiction to sugar and caffeine, and also felt refreshed and energetic after consuming them.

## Discussion

The present study led to a comprehensive understanding of the existing policies and food environment (availability, affordability, accessibility and marketing) in and around schools and colleges from the perspectives of multiple stakeholders, including, canteen operators, teachers, students (schools, colleges), and parents.

### Existing guidelines and directives

Multiple guidelines and directives for creating an enabling food environment in and around educational institutes exist in India [16–20]. The lack of comprehensiveness and plurality of these guidelines and directives made challenging to implement these in their institutions. The recently introduced, Food Safety and Standards (Safe food and balanced diets for children in school) Regulation, 2020 [21] is a step in a positive direction but it only applies to schools. The regulation provides an opportunity to improve the health and wellbeing of school students by ensuring the availability of healthy and safe foods in and around school right from the initial years of life. Food Safety and Standards (Safe food and balanced diets for children in school) Regulation, 2020 [21] envisages to attain Sustainable Development Goal-3 (health and well-being), 4 (quality education)) [31], and also in alignment with the best practices of the developed nation to combat obesity [32–34]. Evidence from various countries has shown that the national school canteen guidelines effectively reduce overweight and obesity rates [35]. The implementation of canteen policy evaluated in countries like Saudi Arabia [36] and Brazil [37] showed that schools did not comply with the policies. Hence to ensure the effective implementation of the regulations in India, specific attention must be given to monitoring and surveillance aspects, which is a key to ensure compliance [21]. Similar to regulation for schools, the need of the hour is to have a regulation for colleges. Given the differential situations in and around Indian colleges with various food outlets, vending machines, fast food restaurants, the policy should be tailored to these contextual aspects.

### Food environment and policies in and around educational institutes

#### Availability and accessibility of foods and beverages

Available packed food and beverage options in canteens of participating schools and colleges were from red category or either HFSS, despite the restrictions on selling these items. Similar to our findings, studies carried out in schools and colleges in several parts of India [38–40] and a narrative review of the nutrition quality of the school canteen in South Asia [41] reported easy and high availability of energy-dense foods and beverages in school’s canteen. It is essential to regulate the availability of HFSS foods and beverages in school environment as children neglect the consequences of these unhealthy items and consume the readily available food within their environment [42]. This indicates weak enforcement of existing guidelines and directives that restrict the availability of HFSS foods and beverages in school [19–21] and college canteens affiliated to the University Grants Commission [20].

Guidelines also suggest categorising the non-standardised proprietary foods and beverages based on the colour coding concept (green, red and yellow), as per their nutritional value, to encourage consumption of healthy foods [16, 17]. Our study revealed that none of the schools and colleges were practicing colour coding methods in their canteens. Globally, interventions in schools have shown that colour coding led to a successfully increased intake of healthy foods and a significant decline in the consumption of HFSS foods during school hours [43]. The findings of our study underscore the need to ensure nutrition labelling with appropriate colour coding, availability of healthy food at affordable prices, to empower the students to make right food choices. There is also a need for a ‘sensitization drive’ through campaigns, nutrition education programmes, seminars for students, teachers and canteen operators to comprehend and understand the benefits of colour coding of food items.

Similarly, easy availability of HFSS foods and beverages through vendors around schools and colleges was another factor that negatively influenced the food environment. The guidelines recommend no sale of HFSS foods to school children within a distance of 50 m [19, 20, 23] to 200 m of a school [16, 19]. Our study revealed that more vendors were observed outside the Private schools than Public schools and throughout the day around the colleges. This highlights the vulnerability of Private school students and college students compared to Public school students. Thus, emphasising the need for implementing the newly introduced regulation [21] to strictly restrict the availability of HFSS foods and beverages within and around schools as defined by the Indian government under the National Multi-sectoral Action Plan for Prevention and Control of NCDs (2017–2022) [44].

#### Affordability of foods and beverages

There was no subsidy available on healthy food options in canteen of schools and colleges. None of the guidelines and directives [16–18, 20] mention any price reduction strategy for promoting the consumption of healthy foods and beverages, nor has it been covered in the recently introduced regulation by FSSAI [21]. In our study, teachers reported that healthy food was subsidised but observations showed that healthy foods and beverages were higher priced than HFSS foods and beverages. Studies conducted in Indian schools [15] also indicated that available nutritious foods in these canteen sold at a higher price [15]. In India, few studies have been done with college students to evaluate the affordability aspect. Although the literature is scanty for both school and college students, the subsidization of healthy food and beverage option in both schools and colleges would be a highly appreciated step in promoting the consumption of healthy foods and beverages. Healthy food generally costs more and are less tasty [45, 46] than HFSS food, and people tend to buy HFSS food due to their low price [47]. The study conducted in the United States to examine the effect of price reduction strategy of healthy foods on the sale of fruits and vegetables among adolescent population showed that fruit sales increased by four fold and vegetables by double during the low-price period [48].

#### Exposure to food marketing

The study reveals that school and college students were exposed to advertisements of foods and beverages both inside and outside their institutes despite restrictions [18, 21]. Unhealthy food advertisements were recognised as one of the significant barriers preventing nutrition promotion and strongly influenced the intake of HFSS foods in young people [49]. In the present study, advertisements outside schools and colleges were primarily of HFSS foods and beverages. The global evidence also highlighted that content of food advertising is mainly on nutrient-poor foods including, confectionery, salty snacks, and sugar-sweetened beverages [50–53] and commercials promoting healthy foods are rarely advertised. Our study also reported events in schools and colleges sponsored by food companies selling foods mostly either HFSS. Research has shown that sponsorship can increase brand awareness, intentions to purchase sponsor products [54, 55] and modify the brand image [56]. In all the directives, marketing and advertisement guidelines for HFSS foods have been specified, but the disciplinary action is lacking [16–21].

The overall findings of our study showed that both school and college children are spending most of their time in an unhealthy food environment. Private school students are more vulnerable in comparison to Public schools due to various factors that influence the food environment like availability of HFSS foods and beverages in the canteen and around schools through vendors, higher density of vendors outside the school, exposure to HFSS advertisements and students’ purchasing power. These factors may contribute to increasing the prevalence of overweight and obesity among children and adolescents of Private schools. This is also evident from previous studies conducted in India by our group that Private school students are at a higher risk of being overweight and obese in comparison to Public school students [57].

The Government of India has taken several steps to mitigate the risk of developing overweight and obesity and improving the food environment, like the launch of the National School Health programme under Ayushman Bharat to promote healthy behaviours among the children [58], FSSAI’s Eat Right Campaign [59] and several other guidelines and directives [16–21]. With the introduction of new regulation [21], regular inspection is a vital move to ensure its compliance and mid-course correction should be suggested to schools keeping into account the barriers faced by schools in enforcing the regulation.

A key strength of the study was the involvement of multiple stakeholders including parents, teachers, students, and canteen operators, to understand the factors attributing to existing obesogenic food environments in and around schools and colleges. The study also employed a mixed-methods approach to validate data from multiple sources and understand the situation extensively, which would not have been possible if only one approach (quantitative/ qualitative) was used. There is a limited amount of research available around the food environment in colleges of India, hence this study is unique in including young adults. There are few limitations as no survey was done with students; the study was restricted to Delhi and NCR, hence limiting the generalizability of study findings. More research is needed to understand the implementation of the new FSSAI regulation, compliance of schools and understand the roadblocks faced by schools in effective implementation of new policies across states and Union Territories of India.

## Conclusion

The findings from our study reveal various determinants influencing the obesogenic food environment in educational institutes in India, despite the supportive guidelines for food service in canteens and food availability outside schools. Thus, underscoring the need to address this challenge through regular monitoring and surveillance of the recently introduced regulation to ensure its compliance and appropriate enforcement strategies.

## Supplementary Information

**Additional file 1.** Study tools.

## Data Availability

The datasets used analysed for the current study are available from the corresponding author on reasonable request.

## Abbreviations

FGD: Focus Group Discussion
FSSAI: Food Safety and Standard Authority of India
HFSS: High in Fat, Salt and Sugar
IDI: In-Depth Interview
NCD: Non-Communicable Diseases
SEAR: South East Asian Region
USD: United States Dollar
WHO: World Health Organization
