# Supplementary material for: Food environment in and around schools and colleges of Delhi and National Capital Region (NCR) in India
Source: BMC Public Health. 2021 Sep 28;21:1767. doi: 10.1186/s12889-021-11778-6 (PMC8477491; doi:10.1186/s12889-021-11778-6)
Supplement: Supplementary file 1 — Additional file 1. Study tools. [file 12889_2021_11778_MOESM1_ESM.docx]

**Guide: Focus Group Discussion with School/college Students**

*School/ College details*

School / College (Identity Code):

Class:

Number of students participated:

Name of Moderator:

Name of Note taker:

Discussion start time:

Discussion end time:

**Guide**

**Reasons for use and consequences of unhealthy foods**

- Why do you think people of your age tend to eat unhealthy food?
- In general, what do you think about the consequences of junk foods/unhealthy food/beverages/ HFSS (foods with high fat, sugar and salt content)?
- How could children of your age be encouraged to eat healthy foods?

**Eating behaviours/practices**

- Do children of your age drink soft-drinks? How often and/or when?
- Do children of your age eat fried foods? How often and/or when?
- Do children of your age consume fruits and vegetables regularly (e.g. everyday)? Why? Why not?
- Do children of your age consume milk and its product regularly (e.g. everyday)? Why? Why not?
- How frequently do you eat outside? What food you prefer to order when you eat outside?
- If you do not bring a lunch from home, where would you prefer to buy food from? What would you prefer to eat?
- What is usual practice of eating out? How often you eat outside food? Which food usually you people like to have and from where?

**School/College Environment and Initiatives**

- What responsibility do you think the school/college should have in order to contribute to healthy eating habits amongst its’ students? How important do you think it is for schools/colleges to address this issue?
- Is there is a canteen in your school/college? What kind of food and beverages are available in your canteen? What do you think about the food and beverages being sold and served in your school/college canteen?
- Do you have an idea about any restriction on the sale of any particular food items/beverages in your school/college canteen?
- Have you ever observed any food or beverages advertisements in your school canteen or anywhere else in school/ /college?
- What do you think about students being allowed to buy food from the vendors outside the school/college gate during lunch break? What is the policy regarding this in your school/college? What kind of foods usually sold by these vendors?

**Influence of food and beverage advertisements**

- Of all the advertisements for foods or beverages (drinks), which one do you like the most? What is the product being advertised? How often have you seen this?
- Where have you seen this advertisement? What did you understand from this message?
- Did that advertisement make you feel like trying the product it advertised?
- Have you ever observed any advertisement and promotion of pre-packaged foods targeted at children around your school/college? Where have you seen this? Which advertisement? What message?

**Barriers and facilitators of healthy eating**

- In your opinion, what could be few reasons that students like you might want to eat healthier foods or beverages?
- In your opinion, what could be few reasons that students like you might not eat healthy foods or beverages?
- What barriers and enablers of healthy behavior can you identify among children?

**CANTEEN ASSESSMENT TOOL FOR SCHOOLS AND COLLEGES**

1. **General Information**
2. School/College ID: ______________________________
3. Name of the interviewee: _________________
4. Name of the school/college: ______________________
5. Role: Canteen Operator □ Member of Canteen Management Committee □
6. Designation at School/college (*in case of teacher being member of Canteen Management Committee*): ___________
7. School/College Type: Public □ Private
8. Geographical area: Delhi □ NCR □
9. Name of the Interviewer: __________________
10. Date of conducting interview: ________

| **S. No.** | **Question** | **Response** | | | |
| --- | --- | --- | --- | --- | --- |
| 1. | Since how long are you running the canteen in this school/college? | _________years  (number of years) | | | |
| 2. | Who manages the canteen services in your school/college? | | | | |
|  | Internal management (School/College Management) | □ | | | |
|  | External management (Contracted to a commercial operator) | □ | | | |
|  | Any other, please specify___________________ | □ | | | |
| 3. | On usual school/college days, how many students avail the canteen services? | _____(Number of students avail canteen services per day) | | | |
| 4. | What time are the canteen services accessible for the students? | | | | |
|  | During school/college hours | □ | | | |
|  | Recess period only | □ | | | |
|  | After school/college hours | □ | | | |
| 5 (A) | Does the school/college have nutritional policy or guidelines for the foods available in the canteen? | | | | |
| a) | Yes, written policy or guidelines | □ | | | |
| b) | Yes, unwritten policy or guidelines | □ | | | |
| c) | No policy or guidelines | □ | | | |
| d) | Do not know | □ | | | |
| 5(B) | If yes, what does the policy/guidelines specify? | | | | |
| a) | Only ban on the sale of foods high in fats in canteen | □ | | | |
| b) | Only ban on the sale of foods high in sugar in canteen | □ | | | |
| c) | Only ban on the sale of foods high in salt in canteen | □ | | | |
| d) | Ban on the sale of all foods high in fats, sugar and salt in canteen | □ | | | |
| e) | Color coding for the Non-standardized proprietary foods (foods prepared in school premises such as *samosa, pakora, noodles* etc.) according to its nutritional value | □ | | | |
| f) | Any other, please specify_________________ | □ | | | |
| 6. | Which of the following foods are available in your canteen? (Tick all that apply) | Yes | No | If yes, Serving Size  (Small/ Medium/ large) | If yes, Price per serving (in INR) |
|  | Chips, fried foods like Potato fries | □ | □ | ________ | ________ |
|  | Sweets (Rasagulla, Gulab jamun,Peda, Kalakand, Jalebi, Imarti, Boondi etc.) | □ | □ | ________ | ________ |
|  | Noodles/Pasta | □ | □ | ________ | ________ |
|  | Pizzas | □ | □ | ________ | ________ |
|  | Burgers | □ | □ | ________ | ________ |
|  | Tikki | □ | □ | ________ | ________ |
|  | Gol gappas | □ | □ | ________ | ________ |
|  | All types of chewing gum and candies | □ | □ | ________ | ________ |
|  | Plain chocolates including dark chocolate | □ | □ | ________ | ________ |
|  | Cakes/pastries | □ | □ | ________ | ________ |
|  | Sweet Buns/ Muffins / Cupcakes | □ | □ | ________ | ________ |
|  | Vegetable sandwiches | □ | □ | ________ | ________ |
|  | Whole Wheat Roti/Paratha stuffed with Seasonal Vegetable | □ | □ | ________ | ________ |
|  | Rice and Dal(Rajma/ chawal/ kadi/ sambhar) | □ | □ | ________ | ________ |
|  | Vegetable Pulao | □ | □ | ________ | ________ |
|  | Idli/Vada/Dosa and Sambhar | □ | □ | ________ | ________ |
|  | Processed namkeens like Aloo bhujia | □ | □ | ________ | ________ |
|  | Vegetable Upma/Uttapam | □ | □ | ________ | ________ |
|  | Fruit/Vegetable salads | □ | □ | ________ | ________ |
|  | Whole Fruits | □ | □ | ________ | ________ |
|  | Samosa/ Pakora/Bread pakora | □ | □ | ________ | ________ |
|  | Vegetable Cutlet | □ | □ | ________ | ________ |
|  | Dhokla/Khandvi | □ | □ | ________ | ________ |
| y) | Biscuits | □ | □ | ________ | ________ |
| z) | Any other , please specify____________________________ | □ | □ | ________ | ________ |
| 7. | Which of the following beverages are available in your canteen? (Tick all that apply) | Yes | No | If yes, Serving Size  (Small/ Medium/ large) | If yes, Price per serving size |
|  | Carbonated beverages (sweetened eg. soft drinks) | □ | □ | ________ | ________ |
|  | Carbonated beverages (unsweetened eg. soda, diet) | □ | □ | ________ | ________ |
|  | Packed juices (sweetened) | □ | □ | ________ | ________ |
|  | Packed juices (no added sugar) | □ | □ | ________ | ________ |
|  | Fresh fruit juice (no added sugar) | □ | □ | ________ | ________ |
|  | Fruit beer | □ | □ | ________ | ________ |
|  | Coconut water | □ | □ | ________ | ________ |
|  | Shikanji/lemonade | □ | □ | ________ | ________ |
|  | Jaljeera | □ | □ | ________ | ________ |
|  | Milk/Flavored Milk | □ | □ | ________ | ________ |
|  | Low fat milk shakes | □ | □ | ________ | ________ |
|  | Probiotic Milk | □ | □ | ________ | ________ |
| m) | Butter Milk/Lassi | □ | □ | ________ | ________ |
| n) | Tea | □ | □ | ________ | ________ |
| 0) | Coffee | □ | □ | ________ | ________ |
| p) | Any other, please specify _______________________________ | □ | □ | ________ | ________ |
| 8(A) | Any special foods/meals served on special occasion/events (eg: sports day, annual day celebration etc.) | | | | |
| a) | Yes | □ | | | |
| b) | No | □ | | | |
| 8 (B) | If yes, what kind of food being served on these special occasions/days? | ______________ | | | |
| 9 | Are you aware about the concept of color coding for the foods to be served in the canteen? | | | | |
| a) | Yes | □ | | | |
| b) | No | □ | | | |
| 10. | What percentage of the foods served in your canteen comprised of green category (e.g: vegetables and legumes, fruits, grain (cereal/ pulses) foods; preferably wholegrain and/or high in fibre, low fat milk, curd, paneer etc.? | | | | |
| a) | 0-25% | □ | | | |
| b) | 26-50% | □ | | | |
| c) | 51-75% | □ | | | |
| d) | 76-100% | □ | | | |
| e) | Don’t know | □ | | | |
| 11. | What percentage of the foods served in your canteen comprised of orange category (e.g: chips, fried foods, ready to eat noodles, Sugar sweetened carbonated and non-carbonated beverages, confectionery items, potato fries, pizza, burgers etc.) | | | | |
| a) | 0-25% | □ | | | |
| b) | 26-50% | □ | | | |
| c) | 51-75% | □ | | | |
| d) | 76-100% | □ | | | |
| e) | Don’t know | □ | | | |
| 12. | What percentage of the foods served in your canteen comprised of yellow category (e.g: Baked vegetable-based snacks, ice creams, milk-based ices and dairy desserts etc.) | | | | |
| a) | 0-25% | □ | | | |
| b) | 26-50% | □ | | | |
| c) | 51-75% | □ | | | |
| d) | 76-100% | □ | | | |
| e) | Don’t know | □ | | | |
| 13. | Does your school/college promote the sale of healthy foods and beverages through any of the following? (Tick all the apply) | Yes | | No | |
| a) | Healthy eating canteen guidelines or regulation | □ | | □ | |
| b) | Pricing guidelines for canteens to encourage sale of healthy food choices at reduced cost | □ | | □ | |
| c) | Training of canteen staff to prepare and serve healthy foods | □ | | □ | |
| d) | Parents are consulted while planning the canteen menu | □ | | □ | |
| e) | Children are consulted while planning the canteen menu | □ | | □ | |
| f) | Teachers are consulted while planning the canteen menu | □ | | □ | |
| 14. | How often the canteen menu gets revised? | ______________ | | | |
| 15. | Which food is most demanded by the students? | ______________ | | | |
| 16. | Which food is least demanded by the students? | ______________ | | | |
| 17. | Which beverage is most demanded by the students? | ______________ | | | |
| 18. | Which beverage is least demanded by the students? | ______________ | | | |

**Teacher Survey: School & College**

**DEMOGRAPHICS**

Name of the school/college: ______________________

Address of the school/college: _____________________

Type: Public □ Private □

Geographic location: Delhi □ NCR □

Your designation: _________________

What is the total number of students in your school/college? ______students

What is the total number of teachers in your school/college? ______teachers

| 1. | Does your school/college ensure the following guidelines/policies/rules/practices with regard to the sale of foods and beverages in canteen? | | **Yes** | **No** |
| --- | --- | --- | --- | --- |
|  | Nutritional guidelines for foods and beverages available in canteen | | □ | □ |
|  | No sale of fried foods (e.g samosa, pakora etc.) in canteen | | □ | □ |
|  | No sale of carbonated and sweetened beverages in canteen | | □ | □ |
|  | No sale of salty foods e.g. wafers, chips etc. in canteen | | □ | □ |
|  | Specific timings for canteen access by students | | □ | □ |
|  | Portion size of available foods | | □ | □ |
|  | Pricing guidelines to encourage sale of healthy food choices at reduced or subsidized price | | □ | □ |
|  | Color coding for the foods according to its nutritional value | | □ | □ |
|  | Sale of “whole foods” like whole grains, unprocessed foods is promoted | | □ | □ |
| j) | Display of healthy eating promotional materials (e.g., posters) in canteen area | | □ | □ |
| 2. | What is the ratio of healthy and unhealthy foods and drinks served in the canteen? | | | |
|  | Almost entirely unhealthy foods and beverages | | □ | |
|  | Almost entirely healthy foods and beverages | | □ | |
|  | Healthy and unhealthy foods and beverages in similar ratio | | □ | |
|  | Healthy foods and drinks are more than unhealthy foods and beverages | | □ | |
|  | Unhealthy foods and drinks are more than healthy foods and drinks | | □ | |
| 3. | No food vendors or hawkers are allowed to sell junk food and/or carbonated drinks within vicinity of 200 meters during the school/college timings (7.00 a.m-4.00 pm). | | □ | □ |
| 4. | Students are allowed to access food outlets around the school/college campus during school/college hours | | □ | □ |
| 5. | Students are allowed to access food outlets around the school/college campus during recess only | | □ | □ |
| 6. | In the last 12 months, any sporting, social or cultural events in your school/ college been sponsored by soft-drink, fast food or confectionary companies? | | □ | □ |
| 7. | If yes, please specify the details____________________________ | |  |  |
| 8. | In the last 12 months, have you seen distribution of newly launched food or the beverages to the school/ college children | □ | □ | |
| 9. | If yes, please specify the name of food/ beverage distributed |  |  | |

**Observation checklist: Schools and colleges**

ID code: _______________________________

Assessment done by: ___________________________

Type: Government □ Private □

State: Delhi □ Delhi-NCR □

School Address:

Date: ______________

Time: _______________

1. Observe and record if there are food vendors or hawkers selling junk food and/or carbonated beverages (food high in fat, salt and sugar) within vicinity of 200 meters during the school/college timings (7.00 a.m-4.00 pm).

1. Yes □
2. No □

If yes, please mention the number of food vendors or hawkers selling junk foods and/or carbonated beverages_____________________(If yes, please click some pictures)

2. During the school/college hours, observe and record if any shops and restaurants selling proprietary foods (e.g: samosas, jalebees, pakoras, burgers, pizzas, aloo-poori, tikki etc.) within vicinity of 200 meters of a school/college to children?

1. Yes □
2. No □

If yes, please mention the number ________________________________________________

3. During the school/college hours (7.00 a.m-4.00 pm), observe and record if you see any students getting food from food outlets or vendors?

1. Yes □
2. No □

If yes, please add details________________________________________________

4. Observe and record if you see any of the following signage in/outside the school/college campus (include all locations such as canteens, classrooms, reception area, sports room, clinics, corridors, library, bulletin boards, office, ground, fences or any other)

| **Signage** | **Yes** | **No** | **Location** | **Visibility (High, medium, low)** |
| --- | --- | --- | --- | --- |
| Food sponsoring company |  |  |  |  |
| Advertisement and promotion of pre-packaged foods targeted at children |  |  |  |  |
| Beverage sponsoring company |  |  |  |  |
|  |  |  |  |  |
|  |  |  |  |  |

5. Do you observe the sale of following foods during your visit to the school/college canteen?

1. Whole fruits Yes □ No□
2. Whole foods (whole grains, unprocessed foods) Yes □ No□
3. Foods high in sugar (carbonated drinks, sweetened fruit juices etc.) Yes □ No□
4. Foods high in fats (samosas, jalebees, pakoras, burgers, pizzas etc.) Yes □ No□
5. High salt foods (e.g wafers, chips etc.) Yes □ No□

6. Observe and record, if proprietary foods (samosas, jalebees, pakoras, burgers, pizzas, aloo-poori, tikki etc.) were categorized based on color coded concept as Green, Yellow and Orange in school/college canteen.

a) Yes □

b) No □

7. Observe and record the percentage of green category foods (e.g: vegetables and legumes, fruits, grain (cereal/ pulses); preferably wholegrain and/or high in fibre, low fat milk, curd, paneer etc. served in the canteen?

a) 0-25% □

b) 26-50% □

c) 51-75% □

d)76-100% □

8. Observe and record the percentage of orange category (e.g: chips, fried foods, ready to eat noodles, sugar sweetened carbonated and non-carbonated beverages, confectionery items, potato fries, pizza, burgers etc.) foods served in the canteen?

a) 0-25% □

b) 26-50% □

c) 51-75% □

d)76-100% □

9. Observe and record the percentage of yellow category (e.g: Baked vegetable-based snacks, ice creams, milk-based ices and dairy desserts etc.) foods served in the canteen?

a) 0-25% □

b) 26-50% □

c) 51-75% □

d)76-100% □

1. Any other observation: _____________________
2. *ADVERTISMENT OUT OF SCHOOL/COLLEGES (UP TO 200 meters)*

| S.No | Distance (upto 200 meters) | Size of advertisement  1=Small  2= Medium  3= Large | Setting  1 = food shop  2 = road  3 = building  4 = bus shelter  5 = nearby metro station  6 =cart/stall | Type of advertisement  1 = Billboard  2=Poster/banner 3 = Freestanding 4 = Painted  5 = Digital/LED  6 = Store merchandising | Number of food products shown (Numeric) If more than one food product used in multiple rows, one row per food product | Brand name | Product name | Promo character | Premium offers |
| --- | --- | --- | --- | --- | --- | --- | --- | --- | --- |
|  |  |  |  |  |  |  |  |  |  |
|  |  |  |  |  |  |  |  |  |  |
|  |  |  |  |  |  |  |  |  |  |
|  |  |  |  |  |  |  |  |  |  |
|  |  |  |  |  |  |  |  |  |  |
|  |  |  |  |  |  |  |  |  |  |
|  |  |  |  |  |  |  |  |  |  |

**PARENT SURVEY: SCHOOL STUDENTS**

1. **BACKGROUND INFORMATION ABOUT YOUR CHILD**

| Name of the child |  | | | |
| --- | --- | --- | --- | --- |
| Gender | Male ⬜ | Female ⬜ | | |
| Type of school | Government ⬜ | Private □ | Kendriya vidyalya ⬜ | |
| Name of School |  | | | |
| Class and Section |  | | | |
| State | Delhi ⬜ | | | Delhi- NCR⬜ |
| Date of Birth (dd/mm/yyyy) |  | | | |
| Age (in completed Years) |  | | | |
| Address (residence) |  | | | |

1. **BACKGROUND INFORMATION ABOUT THE PARENT**
2. Name of Parent (Mother/ Father):
3. Relationship with the child: Mother ⬜ Father ⬜
4. Gender: Male ⬜ Female ⬜
5. Date of Birth: ⬜ ⬜ ⬜ ⬜ ⬜ ⬜ ⬜ ⬜
6. Age (in completed years):
7. Please specify the occupation of child’s mother
8. Where does your child usually get food for his/her lunch on school days?
9. He/she don’t usually eat lunch ⬜
10. Lunch from home ⬜
11. School canteen ⬜
12. Hawkers/rehri wala/ food vendors ⬜
13. Mid-day meal from school ⬜
14. Other, please specify_____________ ⬜
15. If yes to option (7c), In a week, how often does your child eat from school’s canteen?
16. Never ⬜
17. 1-2 times ⬜
18. 3-4 times ⬜
19. 5-6 times ⬜
20. 7 or more times ⬜
21. If yes to any of the option from 8b to 8e, please specify the kind of food item he/she generally prefers to buy from the canteen?
22. Processed or packed foods (chips etc.) ⬜
23. Unpacked foods (sandwich, samosa, pakora) ⬜
24. Ready to eat foods (Noodles etc.) ⬜
25. Sweets (ice cream, pastry, Rasagulla etc.) ⬜
26. Any other, please specify _____________________________ ⬜
27. If yes to option any of the option from 8b to 8e, please specify the kind of beverage he/she generally prefers to buy from the canteen?
    1. Carbonated drinks (sweetened eg. soft drinks) ⬜
    2. Carbonated drinks (unsweetened eg. soda, diet) ⬜
    3. Packed juices (sweetened) ⬜
    4. Packed juices (no added sugar) ⬜
    5. Fresh juices (no added sugar) ⬜
    6. Fresh juices (with added sugar) ⬜
    7. Any other, please specify___________ ⬜

1. How often do you do the following?

|  | **Never** | **Rarely** | **Sometimes** | **Very often** | **Always** |
| --- | --- | --- | --- | --- | --- |
| 1. Limit your child’s intake of sweets and sugary foods (carbonated drinks, juices etc.) | □ | □ | □ | □ | □ |
| 1. Limit your child’s intake of salty foods e.g chips | □ | □ | □ | □ | □ |
| 1. Limit your child’s intake of fried foods like samosa, pakora etc. | □ | □ | □ | □ | □ |
| 1. Encourage your child to eat healthy foods such as fruits, vegetables, whole grains and pulses | □ | □ | □ | □ | □ |
| 1. Limit your child’s intake of chips, chocolates, candies, samosa etc. in between meals | □ | □ | □ | □ | □ |
| 1. Limit your child to order unhealthy foods from restaurant | □ | □ | □ | □ | □ |
| 1. Limit your child to eat from vendors or hawkers | □ | □ | □ | □ | □ |
| 1. Eat meals with your child | □ | □ | □ | □ | □ |
| 1. Prepare healthy options at home | □ | □ | □ | □ | □ |
| 1. Talk about healthy food choices with our child | □ | □ | □ | □ | □ |
| 1. Healthy food rewards for good academic performance | □ | □ | □ | □ | □ |

1. Does your child’s school have any of the following health policy/rules/regulations? Tick all that apply)

| **S.No** | **Policy/Guidelines** | **Yes** | **No** | **Don’t know** | **If yes, written** | **If yes, unwritten** | **If yes, what the policy about? Please mention** |
| --- | --- | --- | --- | --- | --- | --- | --- |
|  | *Food and nutrition policy (e.g* number of nutrition education periods and activities*,*nutrition curriculum etc.) | □ | □ | □ | □ | □ |  |
|  | *Canteen Policy (*nutritional guidelines for foods/beverages available at canteen*,*hygiene monitoring*,*availability of healthy food/beverage options*,*no sale of fried/carbonated/sweetened beverages/salty foods*,*regular monitoring and revision of canteen menu*,*pricing guidelines to encourage sale of healthy foods) | □ | □ | □ | □ | □ |  |
|  | *Lunch box policy (*written guidelines for sending healthy foods in lunch boxes*,*regulation of foods brought from home*,*regular monitoring of lunch box*,*fruit breaks etc.) | □ | □ | □ | □ | □ |  |
|  | *Food vendor policy (*access to food outlets around the school campus during school hours etc.) | □ | □ | □ | □ | □ |  |

1. Which of the following are accessible to your child during school hours? (Tick all that apply):
2. Mid-day meal ⬜
3. Canteen ⬜
4. Food vendors/shops around school campus (7.00A.M TO 4.00 P.M) ⬜
5. If yes to option (13b), do you think the foods served in your child’s school canteen is?
6. Only healthy ⬜
7. Only unhealthy ⬜
8. Both healthy and unhealthy ⬜
9. Do not know ⬜
10. If yes to option (13b), when can students have access to the canteen services in your child’s school (Tick all that apply):
11. All the time during school hours ⬜
12. Only during the lunch break ⬜
13. After school hours also ⬜
14. Any other, please specify______________________ ⬜
15. Which of the following foods are available in your child’s school canteen? (Tick all that apply)

| **S.No** | **Food items** | **Yes** | **No** | **Don’t know** |
| --- | --- | --- | --- | --- |
|  | Chips, fried foods like Potato fries | □ | □ | □ |
|  | Sweets (Rasagulla, Gulab jamun,Peda, Kalakand, Jalebi, Imarti, Boondi etc.) | □ | □ | □ |
|  | Noodles/Pasta | □ | □ | □ |
|  | Pizzas | □ | □ | □ |
|  | Burgers | □ | □ | □ |
|  | Tikki | □ | □ | □ |
|  | Gol gappas | □ | □ | □ |
|  | All types of chewing gum and candies | □ | □ | □ |
|  | Plain chocolates including dark chocolate | □ | □ | □ |
|  | Cakes/pastries | □ | □ | □ |
|  | Sweet Buns/ Muffins / Cupcakes | □ | □ | □ |
|  | Vegetable sandwiches | □ | □ | □ |
|  | Whole Wheat Roti/Paratha stuffed with Seasonal Vegetable | □ | □ | □ |
|  | Rice and Dal(Rajma/ chawal/ kadi/ sambhar) | □ | □ | □ |
|  | Vegetable Pulao | □ | □ | □ |
|  | Idli/Vada/Dosa and Sambhar | □ | □ | □ |
|  | Processed namkeens like Aloo bhujia | □ | □ | □ |
|  | Vegetable Upma/Uttapam | □ | □ | □ |
|  | Fruit/Vegetable salads | □ | □ | □ |
|  | Whole Fruits | □ | □ | □ |
|  | Samosa/ Pakora/Bread pakora | □ | □ | □ |
|  | Vegetable Cutlet | □ | □ | □ |
|  | Dhokla/Khandvi | □ | □ | □ |
|  | Biscuits | □ | □ | □ |
|  | Any other ,please specify____________________________ | □ | □ | □ |

1. Which of the following beverages are available in your child’s school canteen? (Tick all that apply)

| **S.No.** | **Beverages/drinks** | **Yes** | **No** | **Don’t know** |
| --- | --- | --- | --- | --- |
|  | Carbonated drinks (sweetened eg. soft drinks) | □ | □ | □ |
|  | Carbonated drinks (unsweetened eg. soda, diet) | □ | □ | □ |
|  | Packed juices (sweetened) | □ | □ | □ |
|  | Packed juices (no added sugar) | □ | □ | □ |
|  | Fresh fruit juice (no added sugar) | □ | □ | □ |
|  | Fruit beer | □ | □ | □ |
|  | Coconut water | □ | □ | □ |
|  | Shikanji/lemonade | □ | □ | □ |
|  | Jaljeera | □ | □ | □ |
|  | Milk/Flavored Milk | □ | □ | □ |
|  | Low fat milk shakes | □ | □ | □ |
|  | Probiotic Milk | □ | □ | □ |
|  | Butter Milk/Lassi | □ | □ | □ |
|  | Tea | □ | □ | □ |
|  | Coffee | □ | □ | □ |
|  | Any other, please specify _______________________________ | □ | □ | □ |

1. How much do you agree or disagree with the following statements for the development of unhealthy eating practices and habits among children?

| **S.No.** |  | **Strongly Agree** | **Agree** | **Not sure** | **Disagree** | **Strongly Disagree** |
| --- | --- | --- | --- | --- | --- | --- |
|  | Few options of healthy foods and drinks available in school’s canteen | □ | □ | □ | □ | □ |
|  | Foods options available in the school’s canteen lacks variety | □ | □ | □ | □ | □ |
|  | Availability of bigger packets and large serving sizes for foods and beverages | □ | □ | □ | □ | □ |
|  | Healthy food options are not cheaper as compared to unhealthy foods options | □ | □ | □ | □ | □ |
|  | Lack of time to prepare lunch for the children | □ | □ | □ | □ | □ |
|  | Money is easily accessible to children these days | □ | □ | □ | □ | □ |
|  | Parents compensate their lack of availability and time with money or allowing unhealthy eating | □ | □ | □ | □ | □ |

1. Does your child’s school ensure any of the following to promote healthy and nutritious policies and practices?

| **Policy/Guidelines/Practices at your child’s school** | **Yes** | **If yes, how (written or unwritten or any through any other instructions?** | **No** | **Do not know** |
| --- | --- | --- | --- | --- |
| Healthy foods are used to reward students’ good behavior or achievements | □ | **__________** | □ | □ |
| Grade specific nutrition curriculum | □ | **__________** | □ | □ |
| School level nutrition education periods and activities | □ | **__________** | □ | □ |
| School ensures adequate time for students to eat during lunch break | □ | **__________** | □ | □ |
| Healthy food options are available at a reasonable and subsidized price in school canteen | □ | **__________** | □ | □ |
| Availability of nutritional guidelines for foods and drinks served in school canteen | □ | **__________** | □ | □ |
| Restriction on the sale of fried foods (e.g samosa, pakora etc.) in school canteen | □ | **__________** | □ | □ |
| Restriction on the sale of carbonated and sweetened drinks/beverages in school canteen | □ | **__________** | □ | □ |
| Restriction on the sale of salty foods e.g. wafers, chips etc. in school canteen | □ | **__________** | □ | □ |
| Allocated timings for canteen access by students | □ | **__________** | □ | □ |
| Sale of “whole foods” like whole grains, unprocessed foods is promoted | □ | **__________** | □ | □ |
| Regular monitoring and revision of the canteen menu | □ | **__________** | □ | □ |
| Parents are consulted while planning the school canteen menu | □ | **__________** | □ | □ |
| Information is provided to parents about healthy food and eating through promotional material like posters | □ | **__________** | □ | □ |
| Healthy eating education program for parents | □ | **__________** | □ | □ |
| Regular sensitization of children about healthy eating | □ | **__________** | □ | □ |
| Regular monitoring of lunch boxes by teachers | □ | **__________** | □ | □ |
| Fruits breaks for students | □ | **__________** | □ | □ |
| Regulation of foods/drinks brought from home | □ | **__________** | □ | □ |
| Parents are provided with written guidelines about sending healthy food in lunch boxes | □ | **__________** | □ | □ |
| Orientation sessions for parents on school health or canteen policies | □ | **__________** | □ | □ |
| Display of contact numbers of doctors/medical officers in case of any health-related emergencies | □ | **__________** | □ | □ |
| No food vendors or hawkers is allowed to sell junk food and/or carbonated drinks within vicinity of 200 meters during the school timings (7.00 a.m-4.00 pm). | □ | **__________** | □ | □ |
| Access of food outlets by students around the school campus during school hours | □ | **__________** | □ | □ |
| Access food outlets by students around the school campus during recess only | □ | **__________** | □ | □ |

1. How much do you agree or disagree with the following statements?

|  | **Strongly Agree** | **Agree** | **Not sure** | **Disagree** | **Strongly Disagree** |
| --- | --- | --- | --- | --- | --- |
| The school canteen should not sell unhealthy foods (e.g., French fries, samosa etc) | □ | □ | □ | □ | □ |
| The school canteen should not sell unhealthy drinks (e.g., carbonated beverages etc.) | □ | □ | □ | □ | □ |
| Healthy foods sold in the school canteen should be reasonably priced | □ | □ | □ | □ | □ |
| Healthy foods sold in the canteen should be tasty and attractive for students | □ | □ | □ | □ | □ |
| There should be no sale of loose products like samosa, pakora, instant noodles in canteen | □ | □ | □ | □ | □ |
| Food served in the canteen should be color coded to educate children about healthy (green and yellow) and unhealthy foods (orange colour) | □ | □ | □ | □ | □ |
| School should not allow vendors/hawkers upto 200 meters to sell unhealthy foods to children during the school hours | □ | □ | □ | □ | □ |
